# Influence of Calving Ease on In-Line Milk Lactose and Other Milk Components

**DOI:** 10.3390/ani11030842

**Published:** 2021-03-16

**Authors:** Ramūnas Antanaitis, Vida Juozaitienė, Dovilė Malašauskienė, Mindaugas Televičius, Mingaudas Urbutis, Walter Baumgartner

**Affiliations:** 1 Large Animal Clinic, Veterinary Academy, Lithuanian University of Health Sciences, Tilžės Str. 18, LT-47181 Kaunas, Lithuania; dovile.malasauskiene@lsmuni.lt (D.M.); mindaugas.televicius@lsmuni.lt (M.T.); mingaudas.urbutis@lsmuni.lt (M.U.); 2 Department of Animal Breeding, Veterinary Academy, Lithuanian University of Health Sciences, Tilžės Str. 18, LT-47181 Kaunas, Lithuania; vida.juozaitiene@lsmuni.lt; 3 University Clinic for Ruminants, University of Veterinary Medicine, Veterinaerplatz 1, A-1210 Vienna, Austria; walter.baumgartner@vetmeduni.ac.at

**Keywords:** calving ease, in-line, milk lactose

## Abstract

**Simple Summary:**

Calving is a difficult moment in a cow’s life that causes stress, and the ease of calving determines the course of further lactation. The hypothesis of our study was to investigate how the difficulty of calving may influence changes in lactose concentration and other milk components and how well these two factors correlate between each other. We found a statistically significant (*p* < 0.001) negative correlation of calving ease score with milk lactose % (*r* = −0.376) and positive correlation coefficients with milk lactose yield (kg) (*r* = 0.277) as well as milk fat/lactose % ratio (*r* = 0.191). The analysis showed a regular increase (*p* < 0.001) with decreasing calving ease scores for milk electric conductivity and milk somatic cell count.

**Abstract:**

The aim of our study was to determine how the ease of calving of cows may influence changes in lactose concentration and other milk components and whether these two factors correlate with each other. To achieve this, we compared data of calving ease scores and average percentage of in-line registered milk lactose and other milk components. A total of 4723 dairy cows from nine dairy farms were studied. The cows were from the second to the fourth lactation. All cows were classified according to the calving ease: group 1 (score 1)—no problems; group 2 (score 2)—slight problems; group 3 (score 3)—needed assistance; group 4 (score 4)—considerable force or extreme difficulty. Based on the data from the milking robots, during complete lactation we recorded milk indicators: milk yield MY (kg/day), milk fat (MF), milk protein (MP), lactose (ML), milk fat/lactose ratio (MF/ML), milk protein/lactose ratio (MP/ML), milk urea (MU), and milk electrical conductivity (EC) of all quarters of the udder. According to the results, we found that cows that had no calving difficulties, also had higher milk lactose concentration. ML > 4.7% was found in 58.8% of cows without calving problems. Cows with more severe calving problems had higher risk of mastitis (SCC and EC). Our data indicates that more productive cows have more calving problems compared to less productive ones.

## 1. Introduction

Calving is a hazardous moment in a cow’s life that causes stress, not only that, the ease of calving determines the course of further lactation. Dystocia is a prolonged and difficult calving where a veterinary intervention is required. However, the time from the second stage of calf birth (amniotic rupture) to the moment when help should be provided, varies on average from 2 to 3 h [1]. The assessment of the severity of dystocia is not well defined, but most sources provide a dystocia scoring system from 0 to 4 or from 0 to 5 score, where 0 is easy calving and no intervention is needed, and 4–5 is very difficult calving when veterinary help is needed [2]. The ease/difficulty of calving depends on a variety of factors [2]. The main risk factors for difficult calving (dystocia) are associated with proximal or immediate causes such as feto-pelvic disproportion; uterine inertia; fetal malposition; vulval or cervical stenosis; uterine torsion [3,4,5,6,7]. Cows that suffered from dystocia, the risk for various diseases after calving, such as retained placenta, ketosis, metritis, displacement of abomasum, or mastitis increases [2,8]. Data from several studies indicate that dystocia may cause a number of adverse consequences, such as loss of production related to decreased milk yield [9], decreased milk protein, fat and lactose as well as increased somatic cell counts in milk [10]. Problematic calving and subsequent consequences have also been found to affect the fertility of cows negatively [10].

A difficult calving may negatively influence the productivity of cows [11]. Moore et al. [12] found moderately positive correlations between calving and protein, milk and fat yield. In the first part of lactation, a veterinary-assisted dam showed a clear loss in milk yield compared to a nonassisted dam. An interesting finding has been established, that a difficult birth has long-term effects on the production of a calf in later life. The physiological causes or effects influencing a troublesome birth appear to be long-lived. This problem needs acknowledged, and more studies must be undertaken [11].

The ability to record milk lactose concentration using automatic milking systems (AMS) allows its changes to be monitored in real time, several times a day. Thus, it is possible to monitor its changes in various physiological conditions and throughout the period of diseases of cows. Monitoring of lactose concentrations allows for analysis of its relationship with other parameters. An association between milk lactose percentage and cow fertility has been established [13]. Changes in lactose levels in milk have been shown to be a useful indicator for predicting first and second post-calving ovulation [14]. Higher lactose levels are associated with an increased probability of pregnancy [15] as well as a close association of lactose levels with postpartum recovery of luteal function has been reported [16]. Milk electrical conductivity and lactose concentration have been found to be one of the most useful parameters for monitoring and identification of subclinical and clinical mastitis [17]. Since milk lactose concentration decreases during inflammation, it could be used as an indicator for mastitis [17]. The adoption of precision farming technologies on a large scale allows for the daily registration of individual milk and changes of certain milk components that could be used to detect health disorders and start early treatment.

Monitoring of lactose concentrations has become widely used as a parameter for early diagnosis and herd management. An association between milk lactose percentage and cow fertility has been established [13]. Changes in lactose levels in milk have been shown to be a useful indicator for predicting first and second post-calving ovulation [14]. Higher lactose levels are associated with an increased probability of pregnancy [15] as well as a close association of lactose levels with postpartum recovery of luteal function has been reported [16]. Milk electrical conductivity and lactose concentration have been found to be one of the most useful parameters for monitoring and identification of subclinical and clinical mastitis [17]. Since milk lactose concentration decreases during inflammation, it could be used as an indicator for mastitis [17]. The ability to record milk lactose concentration using automatic milking systems (AMS) allows its changes to be monitored in real time, several times a day. Thus, it is possible to monitor its changes in various physiological conditions and throughout the period of diseases of cows. The adoption of precision farming technologies on a large scale allows for the daily registration of individual milk and changes of certain milk components that could be used to detect health disorders and start early treatment.

While researching literature, we found a lack of information on how calving ease affects in-line milk lactose levels and other milk components, them being important tools in cow health and productivity assessment. Our hypothesis was that ease of calving has an influence on the lactose concentrations of milk and that they are closely related. Therefore, we set an aim to evaluate the relationship of calving ease (determined by scoring) and the average percentage of in-line registered milk lactose concentration and other milk components.

## 2. Materials and Methods

### 2.1. Location and Animals

The study was carried out in nine Lithuania dairy farms (from 1 February 2018–7 April 2020) with more than 500 milking cows. A total of 4723 dairy cows from nine dairy farms were studied. The selection of the cows was according to the following criteria: cows had to be first 30 days after calving, having two or more lactations. The cows were kept in a free-stall barn and were fed a total mixed ration (TMR) routinely throughout the year, which was balanced according to their physiological and production needs. Feeding time for the cows took place every day at 06:00 and 18:00 with a total mixed ration for high-producing, multiparous cows. Diets were formulated accordingly to meet or exceed the requirements of a 550 kg Holstein cow producing 35 kg of milk per day according to NRC [18]. The average chemical composition of rations of the nine farms is as follows: dry matter (DM) (%) 48.8; neutral detergent fiber (% of DM) 28.2; acid detergent fiber (% of DM) 19.8; non-fiber carbohydrates (% of DM) 38.7; crude protein (% of DM) 15.8; net energy for lactation (Mcal/kg) 1.6. On average 2 kg per day of concentrate were provided for the cows at the milking robot during milking.

### 2.2. Measurements

Milk traits of all 4.723 cows were studied during the early lactation (from calving to 30 days after calving). 

Cows were milked with Delaval milking robots with free traffic and combined with the fully automated real-time milk analyzer Herd Navigator (DeLaval Inc. Tumba. Sweden). The milking robot automatically takes a representative several milliliters sample of milk from a cow during the milking process. Calving score records were collected during 2018–2020 by trained farms technicians at the same methodology according to Jensen [19]. For the evaluation of the calving score, a 4-point scale was used accordingly: 1—easy unassisted (*n* = 2.264, 47.94%); 2—easy, assisted (*n* = 1.479, 31.31%); 3—difficult, assisted (*n* = 490, 10.37%); 4—difficult, requiring veterinary assistance (*n* = 490, 10.37%). A total of 4723 calvings were assessed. All cows were identified when they start calving according Saint-Dizier and Chastant-Maillard (2015) three stage methodology [20].

The cows were from the second to the fourth lactation. 

Based on the data from the milking robots, during first 30 days after calving every day milk indicators of the cows were recorded: milk yield MY (kg/day), milk fat (MF), milk protein (MP), lactose (ML), milk fat %/lactose % ratio (MF/ML), milk protein %/lactose % ratio (MP/ML), milk urea (MU), and milk electrical conductivity (EC) of all quarters of the udder (according to Televičius et al. [21], milk lactose level during first 30 days after calving (21.2 ± 11.04 days). All cows (*n* = 4.723) were divided into two groups: group 1—lactose < 4.70% (*n* = 2.732, 57.84%); group 2—lactose ≥ 4.70% (*n* = 1.991, 42.16%). Based on the average milk EC level, the cows were divided into four classes: EC < 4.5 mS/cm (*n* = 1.294, 27.11%), EC = > 4.5–5.5 mS/cm (*n* = 2.940, 61.60%), EC > 5.5–6.5 mS/cm (*n* = 477, 9.99%), EC > 6.5 mS/cm (*n* = 62, 1.30%). 

### 2.3. Data Analysis and Statistics

The statistical analysis of data was performed using the SPSS 25.0 programme package (SPSS Inc., Chicago, IL, USA). The normality of data was assessed for all variables using the Kolmogorov–Smirnov test. To obtain a normal distribution, milk somatic cell (SCC) data were transformed into somatic cell score (SCS = log2(SCC/100) + 3). Thus, parametric methods of statistical analysis were applied to all milk indicators. Variables of the descriptive statistics are presented as mean ± standard error of mean (M ± SEM) and 95% confidence interval (CI). When comparing the analyzed milk indicators in terms of milk lactose level, the T-test of independent samples was used. Multiple comparisons of observed mean values with Bonferroni criterion were used to analyze milk characteristics according to the calving ease (CE) scale. The relationships between the studied indicators were examined according to the linear Pearson correlation coefficient (correlation of milk lactose and other milk components), the Spearman correlation (between CE and milk traits) and the χ^2^ test (relation between milk lactose level, other milk components and CE group). For all tests, a probability less than 0.05 was considered significant (*p*-Value < 0.05).

## 3. Results and Discussion 

During the study there was no apparent increase in the number of somatic cells (SCC) in the milk, which averaged 100.02 ± 5.126 thousand/mL milk. The average milk urea concentration (24.16 ± 0.125 mg/dL) and milk fat to protein ratio (1.20 ± 0.003) of the cows corresponded to balanced norms. The average milk yield of all cows during lactation was 28.27 ± 0.118 kg, milk fat 4.08 ± 0.012%, milk protein 3.48 ± 0.006%, milk lactose 4.65 ± 0.004%, average milk EC of all quarters 4.94 ± 0.008 mS/cm, MF/ML 0.88 ± 0.003, MP/ML 0.75 ± 0.001.

### 3.1. Relationship of Calving Ease with Milk Lactose and OtherMilk Components.

In this experiment, a steady linear decrease in milk lactose % with increasing CE scores was found (Table 1). The average milk lactose percentage of cows in the second CE group was lower than in the first group (0.16 %, *p* < 0.001) and higher than in the third (0.04%, *p* < 0.001) and fourth (0.05%, *p* < 0.001) groups. On the other hand, the average lactose content in kg per day increased with increasing CE scores (from 1.21 ± 0.008 to 1.47 ± 0.016 kg, *p* < 0.01). The analysis showed that the MF to ML ratio increased (from 0.85 ± 0.004 to 0.95 ± 0.009, *p* < 0.001) as the cows’ CE scores deteriorated and calving problems increased. The highest mean of the MP/ML ratio was evaluated in the second CE group (0.77 ± 0.002) and the lowest in the fourth group of cows (0.37 ± 0.004, *p* < 0.001).

According to Costa et al., lactose is suggested as a potential health indicator in cows [3]. Lactose is synthesized in the udder from blood glucose absorbed by the basal membrane of mammary epithelial cells [22]. Glycemia and energy balance in cows have a positive correlation with milk lactose [23], especially in high-producing breeds [24]. Lemosquet et al. [24] suggested that post-hepatic blood glucose availability could be a vital indirect regulator of milk yield. Lactose shows potential to be a valid health indicator in cows [25]. 

**Figure 1:**
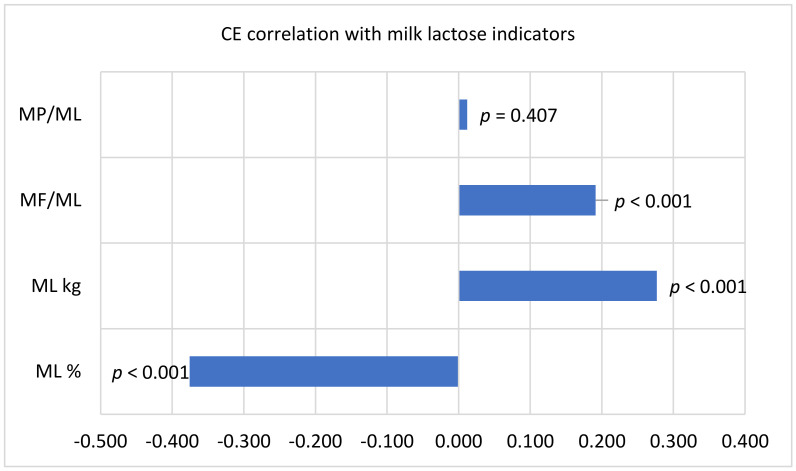
Spearman’s correlation coefficient between cows’ CE scores and milk lactose indicators. ML/MP = milk protein %/lactose % ratio; MF/ML = milk fat %/lactose % ratio; ML = milk lactose.

Based on the data presented in Table 1, the observed trends are confirmed by the calculated correlation coefficients in Figure 1.

We found a statistically significant (*p* < 0.001) negative correlation of CE with cows ML% (*r* = 0.376) and positive correlation coefficients with ML yield (kg; *r* = 0.277) and MF/ML % ratio (*r* = 0.191). 

Furthermore, we registered the correlations between milk lactose and risk of clinical mastitis (based of EC) in early lactation or across the whole lactation, respectively. Costa et al. [26] reported negative correlation between milk lactose and mastitis in the first 150 days in milk. Lactose, EC and their combination together were the most accurate parameters for detection of mastitis in dairy farms equipped with in-line sensors [19]. During mammary tissue inflammation, the osmotic balance is maintained by an increase of Na+ and Cl^−^; in particular, Na^+^ derived from the highly Na^+^-concentrated extracellular environment is the main ion responsible for the increase of the electrical conductivity [3]. Phenotypic correlations between milk lactose and SCC range from −0.15 to −0.66 [27]; in this sense, ML has been widely reported to be one of the most informative parameters used in mastitis diagnosis, other than SCC and milk electrical conductivity [27]. The combined information from ML, SCC and electrical conductivity can be used to provide a precise diagnosis of mastitis at the individual level, the potential of alternative or derived traits (or both) as predictors of udder inflammation [26]. EC and ML were the most reliable indicators of subclinical mastitis (in combination with SCC) and were used to establish predictive patterns of subclinical mastitis in Holstein cows [26]. The decrease of lactose in milk during mastitis can be caused by partly compromised milk lactose synthesis, since the secretory cells are damaged by inflammation, part of the lactose is lost in urine, and a disruption of tight junctions and altered permeability of the basal membrane of the mammary cells that separates blood and milk, mastitis pathogens use available milk lactose to reduce milk lactose and increase lactic acid in milk [3]

Therefore, considering the existing relations between ML and the traits above, lactose and perhaps its ratios with fat, protein or both may be used well as potential biomarkers for metabolic disease in early lactation, as reported by Ederer et al. [28]. 

The experiment showed that ML > 4.7% was found in 58.8% of cows with a calving score of 1, in 27.9% of cows with a calving score of 2, in 26.9% of cows with a calving score of 3, and 23.5% with a score of four points (χ^2^ = 498,970, df = 3, *p* < 0.001).

We detected that milk from cows with higher lactose levels had higher milk protein contents (0.04%) and milk fat content (0.22%) of such cows was lower (*p* < 0.01; Table 2). 

**Figure 2:**
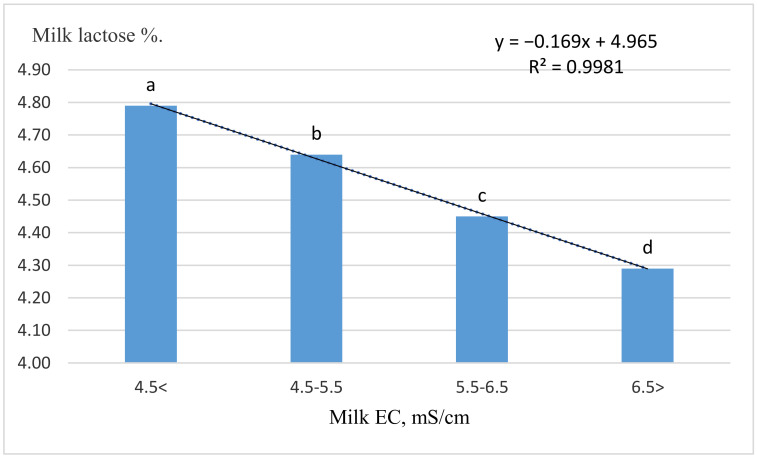
Milk lactose by milk EC level. EC = milk electrical conductivity. ^a, b, c. d^—*p* < 0.01.

**Figure 3:**
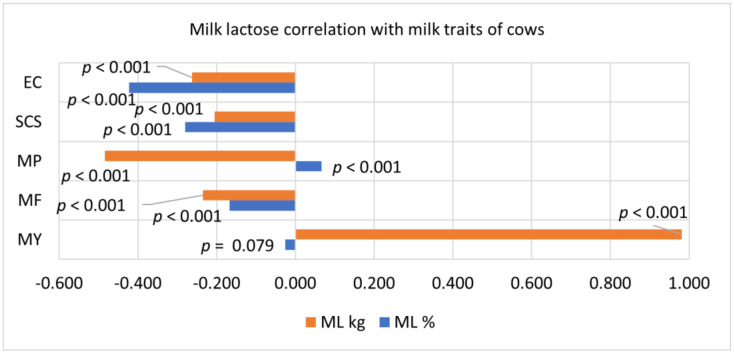
Milk lactose correlation with other milk traits. EC = electrical conductivity of all quarters of the udder; SCS = somatic cell score calculated as log2(number of somatic cells/100) + 3; MP = milk protein; MF = milk fat; MY = milk yield.

The increase in lactose % was closely related to the decrease in the electrical conductivity of milk according to the linear regression equation obtained in Figure 2. Milk electrical conductivity, lactose concentration and their combination together proved to be the most accurate parameters for detection of mastitis in dairy farms equipped with in-line sensors [17]. This is also confirmed by the correlation analysis data summarized in Figure 3. Both milk lactose percentage and lactose content were negatively correlated with milk somatic cells, as was milk EC and fat content. We also found that milk lactose yield (kg) was positively correlated with cow productivity (*p* < 0.001). The dependence of milk yield on lactose concentration and the uptake of glucose from the blood to produce lactose is a metabolic priority [29]. 

### 3.2. Relation of Calving Score with Milk Traits of Cows

Our data indicates that higher yielding cows had more problems while calving than cows with lower milk yield. As calving scores increased from one to four, the average productivity of cows increased by 6.77 kg (*p* < 0.001). Not only that, with increasing CE scores, a regular increase (*p* < 0.001) in milk EC and milk somatic cell count was seen (Table 3).

**Figure 4:**
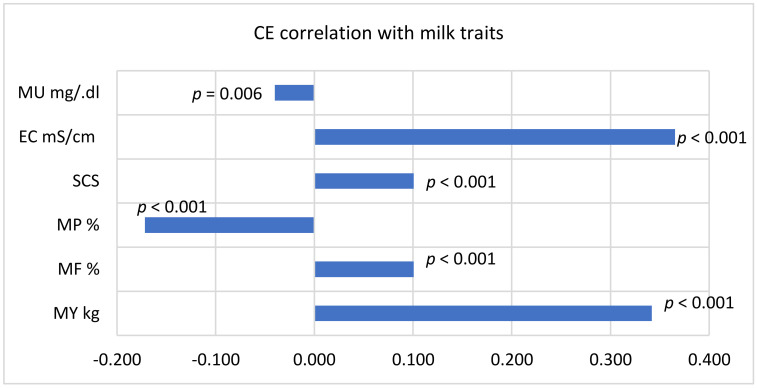
Calving ease score correlation with milk traits of cows. MU = milk urea; EC = milk electrical conductivity of all quarters of the udder; MP = milk protein; MU = milk urea; EC = milk electrical conductivity of all quarters of the udder; SCS = somatic cell score calculated as log2(number of somatic cells/100) + 3; MP = milk protein; MF = milk fat; MY = milk yield.

CE score positively correlated (*p* < 0.001) with MY (*r* = 0.342), EC (*r* = 0.365), MF and SCS (*r* = 0.191) and negatively with MP (*p* < 0.001) and MU (*p* = 0.006; Figure 4). 

A positive association of ML with fertility in the subsequent lactation has been reported by Bastin et al. [30], highlighting increased success in fertility in cows yielding milk with higher ML. Both milk ML and fertility greatly depend on cow energy balance [25], meaning that the relationship between them is likely indirect. On the other hand, Costa et al. [26] reported that genetic correlations were close to zero between ML and a few fertility disorders, namely ovarian cysts and retained placenta. In the last decade, authors have combined ML and milk urea together as an indicator of metabolic health [31]. Ganter et al. [32] stated that due to a negative energy balance at the beginning of lactation, the milk fat content increases, followed by a decrease in milk protein contents, which makes the F/P ratio a good indicator in metabolic disorder identification. Their results state that the amount of lactose in milk, which correlates with the amount of glucose in the blood, can be used to assess the energy balance of cows According to Harjanti and Sambodho [33] the capability of ruminant mammary gland to produce milk is determined by the activity of milk secreting cells and their numbers. Therefore, the milk yield and the concentrations of lactose, protein and fat in milk might be affected by the inflammation level of the mammary gland [34]. There were studies where milk lactose concentrations decreased, and somatic cell counts increased during subclinical and clinical mastitis [22]. Thus, the monitoring of lactose concentrations in milk could be used for identifying cows with mastitis, as with inflammation a clear decrease is seen [22]. Schneeberger and Lynch [35] reported an increase in blood lactose concentration, which is an indicator of mammary epithelium health. 

According our previous study, we found that cows with a higher lactose concentration (≥4.70%) were registered as more active and were at less risk of mastitis (as indicated by lower milk EC and SCC) and metabolic disorders. Low levels of lactose can indicate mastitis (milk SCC ≥ 100 thousand/mL) and metabolic disorders (subclinical ketosis, subclinical acidosis), described by milk F/P. Coulon et al. [34] performed a study to estimate the influence of walking activity on milk production and energy status in dairy farms that housed cows in tie-stall. Dairy cows present signs of sickness in their behavior during mastitis—changes in activity, lying time and feeding behavior have received the most scientific attention [36]. Changes in behavior are suspected to be induced by pain or different unpleasant experiences [37]. SCC increases and milk lactose concentrations decrease during clinical and subclinical mastitis. Milk electrical conductivity, milk lactose and SCC have been widely reported to be some of the most informative traits for mastitis diagnosis [38]. According to a study carried out by Costa et al. [26], there was a genetic correlation between mastitis and milk lactose yield. 

## 4. Conclusions

We found that cows without calving difficulties had higher milk lactose concentrations. ML ≥ 4.7% was evaluated in 58.8% of cows without calving problems and we can suspect that they have a more positive energy balance. Cows with higher calving problems were at higher risk of mastitis (indicated by SCC and EC). Higher yielding cows have more calving problems compared to less productive ones.

## Figures and Tables

**Table 1:** Milk lactose and other milk components by calving ease of cows.

Indicator	CE Score	M	SEM	95% CI	
				Lower Bound	Upper Bound
ML (%)	1	4.75 ^a^	0.005	4.738	4.758
	2	4.58 ^b^	0.006	4.572	4.596
	3	4.54 ^c^	0.011	4.516	4.558
	4	4.53 ^c^	0.011	4.505	4.547
ML (kg)	1	1.21 ^a^	0.008	1.198	1.228
	2	1.37 ^b^	0.009	1.354	1.391
	3	1.46 ^c^	0.016	1.425	1.489
	4	1.47 ^c^	0.016	1.436	1.500
MF/ML	1	0.85 ^a^	0.004	0.842	0.858
	2	0.89 ^b^	0.005	0.876	0.896
	3	0.94 ^c^	0.009	0.926	0.961
	4	0.95 ^c^	0.009	0.929	0.963
MP/ML	1	0.75 ^a^	0.002	0.740	0.748
	2	0.77 ^b^	0.002	0.761	0.770
	3	0.75 ^a^	0.004	0.741	0.757
	4	0.73 ^c^	0.004	0.720	0.736

CE—calving ease: 1—easy, unassisted; 2—easy, assisted; 3—difficult, assisted; 4—difficult, requiring veterinary assistance. ML = milk lactose; MF/ML = milk fat %/ lactose % ratio; MP/ML = milk protein %/lactose % ratio; ^a, b, c^—*p* < 0.05. M—mean; SE—standard of error of the mean.

**Table 2:** Milk composition traits in cows by their lactose level in milk.

Indicator		M	SEM	95% CI	
				Lower Bound	Upper Bound
MF (%)	<4.70%	4.17 ^a^	0.016	4.138	4.200
	≥4.70%	3.95 ^b^	0.019	3.914	3.987
MP (%)	<4.70%	3.46 ^a^	0.007	3.449	3.477
	≥4.70%	3.50 ^b^	0.009	3.481	3.515

MF = milk fat; MP = milk protein; ^a, b^—*p* < 0.01. M—mean; SE—standard of error of the mean.

**Table 3:** Milk traits by calving ease.

Indicator	CE	M	SE	95% CI	
				Lower Bound	Upper Bound
MY (kg)	1	25.546 ^a^	0.160	25.233	25.859
	2	29.850 ^b^	0.198	29.463	30.238
	3	32.056 ^c^	0.343	31.383	32.729
	4	32.315 ^c^	0.343	31.642	32.988
MF (%)	1	4.015 ^a^	0.018	3.980	4.049
	2	4.047 ^a^	0.022	4.005	4.090
	3	4.268 ^b^	0.038	4.194	4.341
	4	4.265 ^b^	0.038	4.191	4.339
MP (%)	1	3.525 ^a^	0.008	3.509	3.540
	2	3.500 ^a^	0.010	3.481	3.519
	3	3.387 ^b^	0.017	3.354	3.420
	4	3.285 ^c^	0.017	3.252	3.318
MU (mg/dL)	1	24.739 ^a^	0.180	24.387	25.092
	2	23.072 ^b^	0.222	22.636	23.508
	3	24.486 ^a^	0.386	23.728	25.243
	4	24.461 ^a^	0.386	23.704	25.219
EC (mS/cm)	1	4.761 ^a^	0.011	4.739	4.783
	2	4.957 ^b^	0.014	4.930	4.984
	3	5.245 ^c^	0.024	5.198	5.292
	4	5.417 ^d^	0.024	5.370	5.464
SCS	1	1.857 ^a^	0.010	1.837	1.876
	2	1.934 ^b^	0.012	1.909	1.958
	3	1.937 ^b^	0.021	1.895	1.979
	4	1.941 ^b^	0.021	1.899	1.983

MY = milk yield; MF = milk fat; MP = milk protein; MU = milk urea; EC = milk electrical conductivity of all quarters of the udder; SCS = somatic cell score calculated as log2(number of somatic cells/100) + 3; ^a, b, c, d^—*p* < 0.05. M—mean; SE—standard of error of the mean.

## Data Availability

The data presented in this study are available within the article.
